# AtCRY2 Negatively Regulates the Functions of AtANN2 and AtANN3 in Drought Tolerance by Affecting Their Subcellular Localization and Transmembrane Ca^2+^ Flow

**DOI:** 10.3389/fpls.2021.754567

**Published:** 2021-11-23

**Authors:** Ting Liu, Leyan Du, Qiushi Li, Jingda Kang, Qi Guo, Shilin Wang

**Affiliations:** ^1^College of Teacher Education, Hebei Normal University, Shijiazhuang, China; ^2^College of Life Sciences, Hebei Normal University, Shijiazhuang, China; ^3^Root Biology Center, College of Natural Resources and Environment, South China Agricultural University, Guangzhou, China; ^4^College of Agriculture, South China Agricultural University, Guangzhou, China

**Keywords:** drought tolerance, photoperiod, calcium, annexin, cryptochrome

## Abstract

Annexins are a multifunctional class of calcium-binding proteins in plants, and their physiological functions and regulation in response to drought stress remain to be elucidated. Here, we found that AtANN2 and AtANN3 conferred to drought tolerance under short-day and long-day conditions, respectively. Under their functional photoperiod, *AtANN2* and *AtANN3* gene expression was enhanced in the mannitol-treated roots, and their encoded proteins were rapidly targeted to the plasma membrane, and mediated significant Ca^2+^ flows across the plasma membrane. Cryptochromes as photoreceptors can not only sense the photoperiod and regulate ion channels on the plasma membrane to influence ion flow but also induce downstream physiological responses. AtCRY2 repressed the functions of AtANN2 and AtANN3 by affecting their plasma membrane localization and inhibited AtANN2- and AtANN3-dependent transmembrane Ca^2+^ flow in response to drought stress. Taken together, these results uncover a mechanism linking Annexins-AtCRY2 to transmembrane Ca^2+^ flow and resulting in enhanced drought tolerance in *Arabidopsis*.

## Introduction

Plant cells trigger a series of signal transduction pathways to regulate physiological responses to adapt to environmental changes (Bechtold, 2018; Gong et al., 2020; Lamers et al., 2020). The plasma membrane serves as the interface between the cell and the environment for the exchange of nutrients and signals. An external signal is sensed by a sensor on the plasma membrane, and then a second messenger is stimulated through transmembrane signal transduction to induce changes in the activity of functional proteins in the cell to regulate physiological responses. Ca^2+^ is an important second messenger widely found in eukaryotic signaling pathways (Kudla et al., 2018). Intracellular Ca^2+^-binding proteins are the targets of calcium signaling, and to date, approximately 200 Ca^2+^-binding proteins have been identified (Bechtold, 2018).

Annexins are a multifunctional class of calcium-dependent phospholipid-binding proteins that can be converted from cytoplasmic soluble proteins to membrane-bound proteins upon binding Ca^2+^ (Davies, 2014). Annexins can act as sensors sensing calcium signals early in the environmental signaling pathway and also trigger the generation of specific downstream calcium signals (Baucher et al., 2012). The study of the physiological functions, environmental regulators, and signal transduction of annexins in plant cells is currently in its infancy. At the cellular level, annexins are localized in the plasma membrane, vesicle membrane, Golgi apparatus and its derived vesicles, chloroplasts, nucleus, glyoxysomes, and mitochondria (Konopka-Postupolska and Clark, 2017). *Arabidopsis* annexins AtANN1 and AtANN2 localized in vesicles can affect vesicle transport and sRNA stability (He et al., 2021), and AtANN1 localized on the plasma membrane can build ion channels (Liu et al., 2021). At the tissue level, the gene expression of annexins is tissue specific, with high expression in secretory cells, such as outer root cap cells, epidermal cells, and pollen tubes (Scheuring et al., 2011). The site of expression is functionally relevant, for example, AtANN1 and AtANN2, which are localized in the root, are involved in post-phloem sugar transport (Wang et al., 2018). At the level of environmental regulators, annexins may be involved in the physiological responses of plants to adverse stress, such as metal stress (Tuomainen et al., 2010), cold stress (Liu et al., 2021), drought stress (Konopka-Postupolska et al., 2009), and salt stress (Ma et al., 2019).

Light often acts as an important environmental factor. The photoperiod, light intensity, and light quality can all affect plant growth, development, and stress tolerance responses (Liu et al., 2020; Yang et al., 2020; Roeber et al., 2021). Light acts through photoreceptors in plant cells, and phytochromes (PHYs) and cryptochromes (CRYs) are the main photoreceptors (Marzi et al., 2020). CRYs can not only sense upstream light signals (Fantini and Facella, 2020) and regulate ion channels and proton pumps on the plasma membrane to influence ion flow (Fogle et al., 2015) but also induce downstream physiological responses (D’Amico-Damiao and Carvalho, 2018). Whether photoreceptor-regulated ion channels include annexins has not been reported, and which physiological responses are involved in annexin-mediated Ca^2+^ flow remains to be investigated.

Recent studies investigating the *Arabidopsis* annexin family focused on the most abundantly expressed annexin, i.e., *AtANN1*. AtANN1 constructs plasma membrane Ca^2+^ channels and is involved in salt and drought stress processes (Laohavisit et al., 2012; Richards et al., 2014; Zhao et al., 2019; Mohammad-Sidik et al., 2021). Whether *AtANN1*-regulated stress responses are related to ion transport requires further investigation. In this work, we found that the photoperiod regulates the physiological function, protein localization, and calcium channel activity of AtANN2 and AtANN3. AtCRY2 is proposed to repress the function of AtANN2 and AtANN3 by affecting their plasma membrane localization and inhibiting AtANN2 and AtANN3-dependent transmembrane Ca^2+^ flow in response to drought stress.

## Materials and Methods

### Materials

The plant material used was *Arabidopsis (Arabidopsis thaliana)*. *atann2* (*AT5G65020*: *SALK_054223*) and *atann3* (*AT2G38760*: *SALK_082344*) were purchased from the Arabidopsis Biological Resource Center (ABRC). The corresponding T-DNA insertion mutants were analyzed by PCR using T-DNA primers and gene-specific primers. For identification at the RNA level by RT-PCR using gene-specific primers, *AtACTIN2* (*AT3G18780*) was used as internal control. The primers are listed in Supplementary Table 1.

The native promoters and coding sequence (CDS) of *AtANN2*, *AtANN3*, and *AtCRY2* (*AT1G04400*) were synthesized and cloned into *pCAMBIA1300-nos* by Sangon Biotech (Shanghai) Co., Ltd. Then, the vector was transformed into these null mutants to generate the complementary lines (*AtANN2-COM* and *AtANN3-COM)*. The CDSs were cloned into *pSuper1300-GFP*, and the vector was also transformed into Col-0 to construct the overexpression lines (*AtANN2-OE* and *AtANN3-OE)*.

### Growth Conditions

*Arabidopsis* seeds were protected from light for 3 days at 4°C and then incubated under different photoperiods. The long-day conditions were as follows: 16 h light/8 h dark, light intensity 100 μmol m^–2^ s^–1^, temperature 22°C light/18°C dark, and relative humidity 70%. The short-day conditions were as follows: 8 h light/16 h dark, light intensity of 200 μmol m^–2^ s^–1^, temperature of 22°C light/18°C dark, and relative humidity of 70%.

The plants used in the growth phenotype assays in response to drought stress were grown in soil. The plants used in the gene expression, β-glucuronidase (GUS) staining, subcellular localization, and transmembrane Ca^2+^ flow assays were grown in 1/2 Murashige and Skoog (MS) medium and 1% (w/v) agar.

### Growth Phenotype Assay

For the drought tolerance tests, long-day conditions included 9 days of drought treatment after 3 weeks of normal plant growth, and short-day conditions included 9 days of drought treatment after 4 weeks of normal growth. The mean number of senescing leaves per plant of different genotypes was counted (*n* = 50).

### Gene Expression Analysis

Quantitative real-time PCR analysis (qRT-PCR) was performed to analyze the gene expression levels. *AtACTIN2* was used as internal control, and the RNA extraction was performed using the RNAprep Pure Plant Kit (Tiangen). cDNA was obtained by the reverse transcription of 2 μg of extracted RNA using iScript^TM^ gDNA Clear cDNA (Bio-Rad). qRT-PCR was performed using a PCR system according to the instructions of SYBR Premix EX Taq (Takara). The PCR program was applied by a StepOne Plus Real Time PCR system (QuantStudio 6 Flex, ABI). Three biological replicates were performed per sample.

### *GUS* Staining

The native promoters of *AtANN2*, *AtANN3*, and *AtCRY2* were synthesized and cloned into *pCAMBIA1391-GUS* by Sangon Biotech (Shanghai) Co., Ltd. The *AtANN2promoter*:*GUS* and *AtANN3promoter*:*GUS* transgenic plants were obtained to observe *GUS* expression in the plants. The plant tissues were incubated in rinse solution [50 mM Na_2_HPO_4_, 50 mM NaH_2_PO_4_,0.5 mM K_3_Fe(CN)_6_, and 0.5 mM K_4_Fe(CN)_6_⋅3H_2_O] for 5 min and then transferred to stain solution [50 mM Na_2_HPO_4_, 50 mM NaH_2_PO_4_,0.5 mM K_3_Fe(CN)_6_,0.5 mM K_4_Fe(CN)_6_⋅3H_2_O, 10 mM EDTA Na_2_, 1% Triton-100 and 2 mM X-Gluc] for incubation for 12 h at 37°C. The plants were eluted two- to three-fold using 95% ethanol at 65°C for 12 h. After decolorization, the specimens were observed under a stereomicroscope (Leica DVM6).

### Subcellular Localization

Transient expression of AtANN2-GFP and AtANN3-GFP was performed by Wuhan Edgene Bio-Tech. (Wuhan) Co., Ltd. using the leaf cell protoplasts. *Arabidopsis* leaves of 3-week-old plants were selected, soaked in enzyme solution [1.5% Cellulase R10,0.75% Macerozyme R10, 600 mM mannitol, 10 mM MES (pH 5.7), and 0.04% 2-hydroxy-1-ethanethiol] for 3 h, and evacuated at 23°C. The samples were filtered into Eppendorf tubes with 40-μm nylon gauze and centrifuged at 400 rpm for 5 min at 4°C. The supernatant was discarded, the samples were washed twice with pre-chilled W5 solution [154 mM NaCl, 125 mM CaCl_2_, 2 mM KH_2_PO_4_, and 2 mM MES (pH 5.7)], W5 solution was added for suspension, and then, the samples were allowed to stand for 30 min at 4°C. Next, MMG solution [400 mM mannitol, 15 mM MgCl_2_.6H_2_O, and 4 mM MES (pH 5.7)] was added for suspension, and then microscopic examination was performed, with 20∼40 protoplasts per field of view obtained under 40 × magnification. The CDSs of *AtANN2* and *AtANN3* were synthesized by Sangon Biotech (Shanghai) Co., Ltd., cloned into the vector *pEGOEP_35__*S*_-H-GFP*, and transformed into the leaf cell protoplasts of Col-0 and *AtCRY2-OE*. Subsequently, 10 μl of target gene plasmid, 100 μl protoplasts, and 110 μl 40% PEG4000 solution were slowly mixed upside down, and then, the mixture was placed in a 22.5°C water bath for 15∼20 min. W5 solution was added to dilute the protoplasts, and the samples were mixed well to terminate the reaction. The samples were centrifuged at 400 rpm for 5 min at 4°C. The supernatant was discarded, and the samples were washed twice with pre-chilled W5 solution. The samples were incubated at 23°C under low light overnight and then observed and photographed under a laser confocal microscope (Olympus FV3000) at an excitation wavelength of 488 nm and an emission wavelength of 510∼530 nm.

The subcellular localization of the AtANN2-GFP and AtANN3-GFP fusion proteins was observed after 300 mM mannitol treatment using 10-day-old transgenic plants under different photoperiods, and GFP signals were observed under a laser confocal microscope (Olympus FV3000).

### Glutathione S-Transferase Pull-Down Assay

For the GST pull-down assay, the coding sequences of *AtANN2*, *AtANN3*, and *AtCRY2* were synthesized by Sangon Biotech (Shanghai) Co., Ltd. The GST pull-down assay was performed by Wuhan Edgene Bio-Tech. (Wuhan) Co., Ltd. *AtCRY2* was cloned into the vector *pET28a*(+), and *AtANN2* or *AtANN3* was cloned into the vector *pGEX-6P-1*. Proteins expressed in *Escherichia coli* were purified as previously described (Cui et al., 2018).

### Bimolecular Fluorescence Complementation

A bimolecular fluorescence complementation (BiFC) assay was performed by Wuhan Edgene Bio-Tech. (Wuhan) Co., Ltd. *AtCRY2* was cloned into the vector *pEGOEP_2__*X*__35__*S*_-cYFP*, and *AtANN2* or *AtANN3* was cloned into the vector *pEGOEP_2__*X*__35__*S*_-nYFP*. The two plasmids were co-transformed into *Arabidopsis* leaf cell protoplasts. The samples were incubated for 16 h in the dark at 23°C. The fluorescence signals were observed using a microscope (Olympus FV3000) at an excitation wavelength of 488 nm and an emission wavelength of 510–530 nm. The parameters were consistent for all samples.

### Measurement of Net Ca^2+^ Fluxes in the Root Using an NMT System

All plates were oriented vertically and maintained in the chamber described above for 5 days. After 5 days, seedlings germinated on 1/2 MS medium were transferred to the test solution [0.1 mM KCl, 0.1 mM CaCl_2_, 0.1 mM MgCl_2_, 0.5 mM NaCl, 0.3 mM MES, and 0.2 mM Na_2_SO_4_, pH 6.0] 30 min for balancing. The Ca^2+^ fluxes of the main root meristematic zone were measured non-invasively by using Non-invasive Micro-test Technology NMT System [NMT100 Series, YoungerUSA LLC, Amherst, MA 01002, United States; Xuyue (Beijing) Sci. & Tech. Co., Ltd., Beijing, China and imFluxes V2.0 (YoungerUSA LLC, Amherst, MA 01002, United States) Software]. The Ca^2+^-microsensor (2–4 μm aperture, XYPG120-2; Xuyue Sci. and Tech. Co., Ltd) was pulled and silanized using glass micropipettes after being filled with a backfill solution of 100 mM CaCl_2_. The backfill solution was pushed into the tip and occupied a length of approximately 1 cm from the tip. The plant materials were collected after the seedlings were exposed to the test solution of 300 mM mannitol or the control system for 10 min. The data were collected every 6 s during the 3 min pretreatment period and 10 min measuring period. These data were obtained from approximately five roots per genotype. The positive values in the figures represent a cation efflux or anion influx.

### Reproducibility and Statistics

All experiments were repeated at least three times. The statistical analyses were indicated in each figure legend and performed using the programs SigmaPlot 12.0 and SigmaStat 3.5. Different letters indicate significant differences with each treatment at the *p* < 0.05 level. Statistical analyses were performed by one-way or two-way ANOVA, followed by Tukey’s multiple comparisons test.

## Results

### Functional Regulation of AtANN2 and AtANN3 in Drought Tolerance by Photoperiod

**FIGURE 1:**
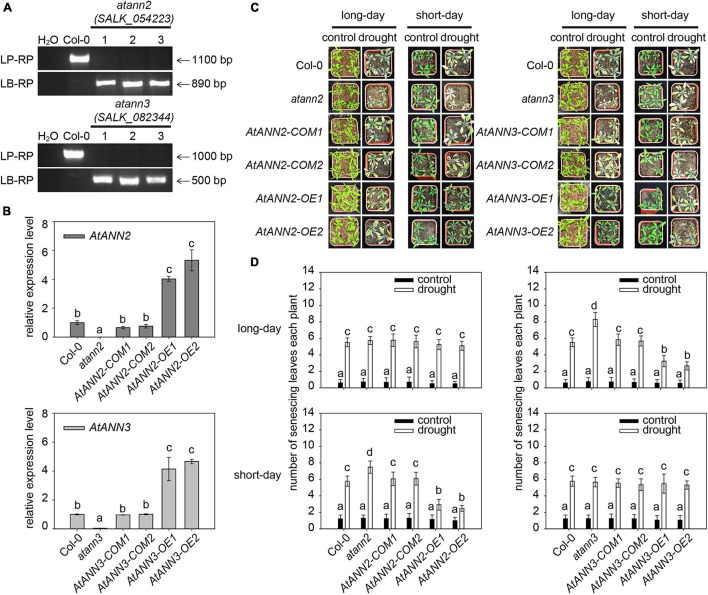
Functional regulation of AtANN2 and AtANN3 in drought tolerance by photoperiod. **(A)** Isolation and identification of *AtANN2* and *AtANN3* T-DNA insertion mutants at the DNA level. **(B)** Identification of *AtANN2* and *AtANN3* T-DNA insertion mutants at the RNA level by qRT-PCR. **(C)** Plant growth phenotype (senescing leaves) of genotypes in response to drought stress under long- or short-day conditions. **(D)** Statistical analysis of senescing leaves of the genotypes shown in Figure 1C. The mean number of senescing leaves per plant was counted (*n* = 50). Different letters indicate significant differences with each treatment at *P*<0.05 level.

To investigate the functions of AtANN2 and AtANN3 in the drought stress response, their corresponding T-DNA insertion mutants were isolated (Figure 1A). In wild type Col-0, a specific band ranging from 1,000 to 1,100 bp in size was amplified using a gene-specific primer (LP and RP), while no band was detected using the T-DNA primer (LB and RP). In *atann2* (*salk_054223*) and *atann3* (*salk_082344*), the T-DNA primer amplification yielded bands with sizes of 890 and 500 bp, respectively, indicating the presence of T-DNA insertion into the target genomic regions. The qRT-PCR analysis showed that the expression levels of *AtANN2* and *AtANN3* genes were completely disrupted in the null mutants *atann2* and *atann3*, and were able to be recovered in the complementary lines *AtANN2-COM* and *AtANN3-COM* to the same level as those in Col-0. In overexpression lines *AtANN2-OE* and *AtANN3-OE*, the expression levels of *AtANN2* and *AtANN3* genes were enhanced up to four- to five-fold (Figure 1B).

The plant growth phenotypes of the obtained mutants and transgenic lines in response to drought stress were determined under long and short-day conditions (Figures 1C,D). The number of senescing leaves of plants was significantly increased by drought treatment compared to that in the untreated plants. Under short-day condition, compared to that in wildtype Col-0 (5.78), a higher number of senescing leaves was determined in the *atann2* mutant (7.48), while less numbers of senescing leaves in the *AtANN2-OE* (*OE1* and *OE2*) lines (2.48–2.92), suggesting that AtANN2 plays a role in drought tolerance. As a control, the *AtANN2-COM* (*COM1* and *COM2*, 6.06–6.08) lines showed no difference from that of Col-0. Under long-day condition, however, the contribution of AtANN2 to drought tolerance was not determined as all genotypes showed the similar numbers of senescing leaves under drought treatment. Unlike AtANN2, the AtANN3-related genotypes showed different responses to drought stress under long-day condition rather than short-day condition. Compared to that in wildtype Col-0 (5.52), a higher number of senescing leaves was determined in the *atann3* mutant (8.30), while less numbers of senescing leaves in the *AtANN3-OE* (*OE1* and *OE2*) lines (2.66∼3.24), suggesting that AtANN3 plays a role in drought tolerance under long-day condition. These results indicated the functions of AtANN2 and AtANN3 in drought tolerance dependent on the photoperiod.

### Gene Expression Patterns of *AtANN2* and *AtANN3* in *Arabidopsis* Seedlings Under Normal Condition

**FIGURE 2:**
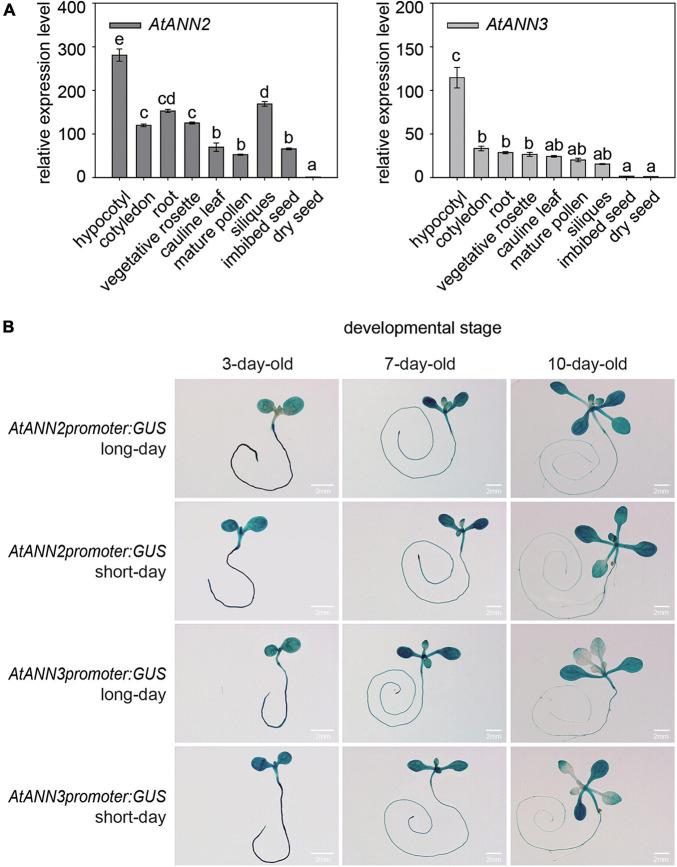
Gene expression patterns of *AtANN2* and *AtANN3* in *Arabidopsis* seedlings under normal condition. **(A)** Gene expression patterns in different tissues using qRT-PCR analysis. **(B)** Gene expression patterns in developmental stages under long-day and short-day conditions using *GUS* reporter gene transgenic plants. Scale bar = 2 mm.

By qRT-PCR analysis, the *AtANN2* gene was found to be expressed mainly in hypocotyls, roots, vegetative rosettes, and siliques, and the *AtANN3* gene was mainly expressed in hypocotyls, cotyledons, and roots (Figure 2A). Using *GUS* reporter gene transgenic plants, *AtANN2* and *AtANN3* promoter activities were mainly expressed in the roots and cotyledons of 3-day-old seedlings, slightly increased in the root tips and shoots of 7-day-old seedlings, and further decreased in the roots and shoots of 10-day-old seedlings (Figure 2B). These expression patterns of *AtANN2* and *AtANN3* did not differ between long-day and short-day conditions.

### Expression Patterns of *AtANN2* and *AtANN3* in Response to Mannitol Treatment Under Different Photoperiods

**FIGURE 3:**
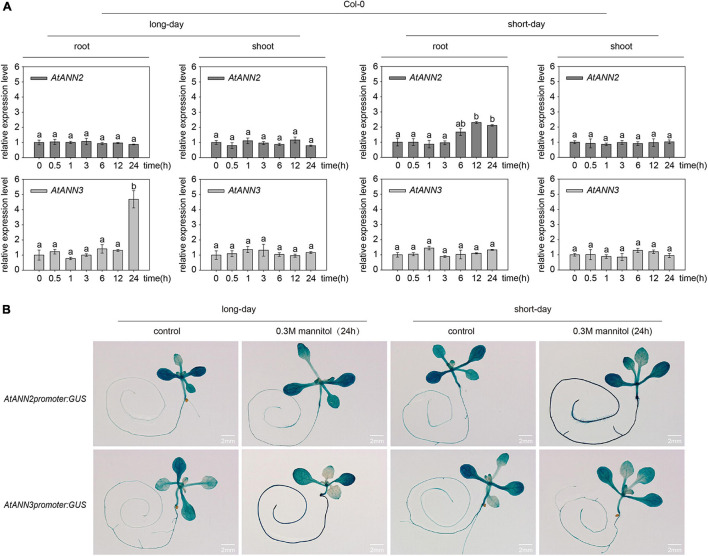
Expression patterns of *AtANN2* and *AtANN3* in response to mannitol treatment under different photoperiods. **(A)** Relative expression levels of *AtANN2* and *AtANN3* in 300 mM mannitol-treated plants (Col-0) for 0, 0.5, 1, 3, 6, 12, and 24 h using qRT-PCR. Different letters indicate significant differences with each treatment at *p*<0.05 level. **(B)** Induction of *GUS* expression in Col-0 roots in response to 300 mM mannitol treatment for 24 h under different photoperiods. Scale bar = 2 mm.

The relative expression levels of *AtANN2* and *AtANN3* were determined in plants treated with 300 mM mannitol (Col-0) for 0,0.5, 1, 3, 6, 12, and 24 h using qRT-PCR (Figure 3A). Compared to the control treatment (0 h), no difference was found in the relative expression levels of *AtANN2* and *AtANN3* in the shoots within 24 h of treatment, while *AtANN2* expression in the roots was increased to 2.30- and 2.11-fold after 12 and 24 h of treatment in the seedlings grown under short-day conditions, respectively. In contrast, *AtANN3* expression in the roots was increased to 4.68-fold after 24 h of treatment in seedlings grown under long-day conditions. *AtANN2* promoter activity was elevated in roots after 24 h of treatment under short-day condition, while *AtANN3* promoter activity was elevated in the roots of treated plants under long-day condition (Figure 3B). These results demonstrated that the photoperiod regulated the expression patterns of *AtANN2* and *AtANN3* in roots in response to mannitol treatment.

### Regulation of the Subcellular Localization of AtANN2 and AtANN3 in Response to Mannitol Treatment Under Different Photoperiods

**FIGURE 4:**
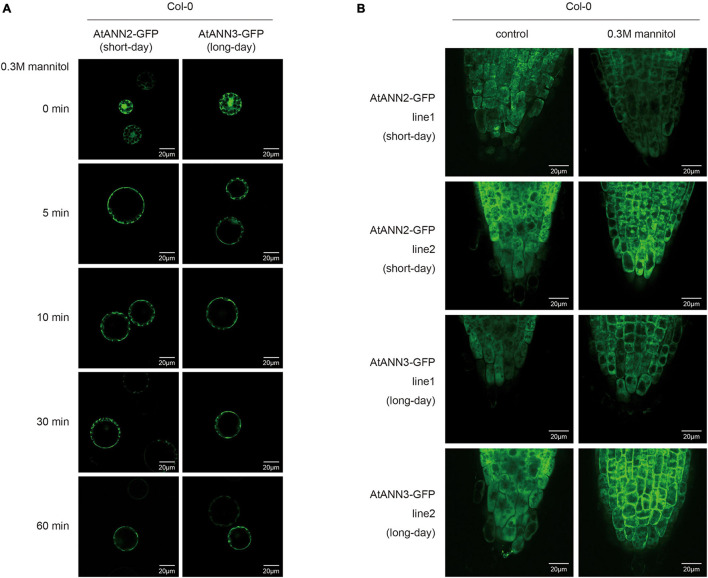
Regulation of the subcellular localization of AtANN2 and AtANN3 in response to mannitol treatment under different photoperiods. **(A)** Subcellular localization of the AtANN2-GFP and AtANN3-GFP fusion proteins in response to mannitol treatment for 0, 5, 10, 30, and 60 min using leaf cell protoplasts of Col-0 plants grown under different photoperiodic conditions. Scale bar = 20 μm. **(B)** Subcellular localization of AtANN2-GFP and AtANN3-GFP fusion proteins using the transgenic lines (line1 and line2). 10-day-old plants under different photoperiods were treated with 300 mM mannitol for 5 min and GFP signals were observed under a confocal laser-scanning microscope. Scale bar = 20 μm.

Leaf cell protoplasts of Col-0 plants grown under different photoperiodic conditions were used to investigate the subcellular localization of the AtANN2-GFP and AtANN3-GFP fusion proteins in response to mannitol treatment for 0, 5, 10, 30, and 60 min (Figure 4A and Supplementary Figure 2A). Under normal conditions, AtANN2- and AtANN3-dependent GFP signals were widely expressed within the cells, not only at the plasma membrane. In the protoplasts of the seedlings cultured under short-day, but not long-day conditions, the AtANN2-dependent GFP signals rapidly targeted to the plasma membrane after 5 min of mannitol treatment. However, regarding AtANN3-GFP, similar subcellular localization changes in response to mannitol treatment were only observed in protoplasts under long-day condition.

These regulatory patterns of the AtANN2 and AtANN3 proteins were further analyzed using transgenic lines expressing *AtANN2-GFP* and *AtANN3-GFP* under their own native promoters (Figure 4B and Supplementary Figure 2B). Again, the cytosolic localization of AtANN2-GFP and AtANN3-GFP rapidly targeted to the plasma membrane in the roots of plants treated with mannitol for 5 min grown under short day condition and long-day condition, respectively. Thus, in response to mannitol treatment, the AtANN2 and AtANN3 proteins could target to the plasma membrane, and this regulatory process was regulated by the photoperiod.

### Regulation of the Transmembrane Ca^2+^ Flow of AtANN2 and AtANN3 in Response to Mannitol Treatment Under Different Photoperiods

**FIGURE 5:**
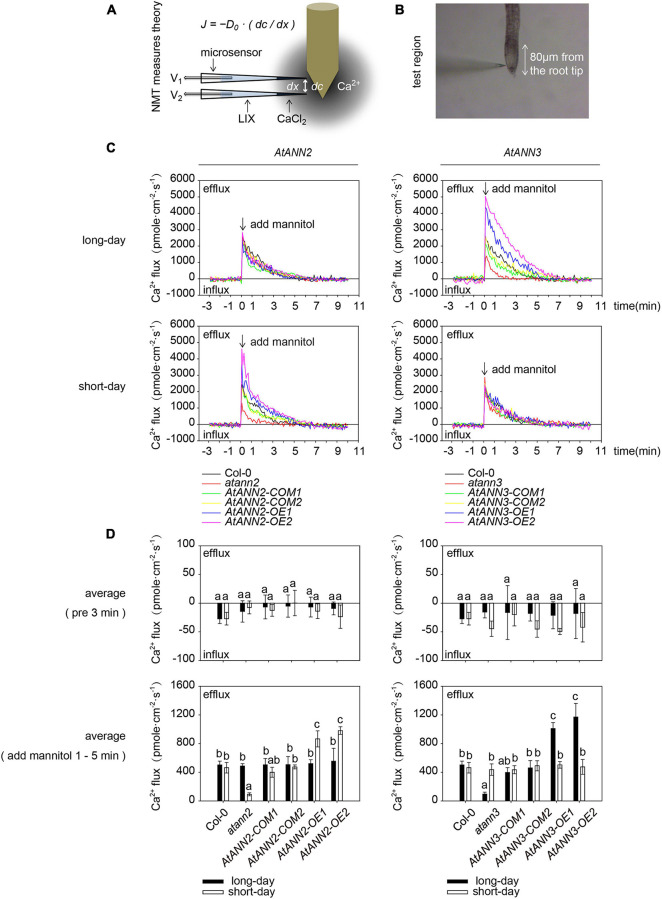
Regulation of transmembrane Ca^2+^ flow by AtANN2 and AtANN3 in response to mannitol treatment under different photoperiods. **(A)** Theory of the NMT system. According to a predefined fashion, the concentration gradient of specific ions was measured between two points near the sample by using an ion microsensor. The ionic fluxes were calculated based on Fick’s law of diffusion as follows: *J* = −*D*_0_⋅(*dc*/*dx*), where *J* is the ion flux (unit: pmole⋅cm^–2^⋅s^–1^), *dc* is the concentration gradient, *dx* is the distance between the two points, and *D*_0_ is the diffusion constant. **(B)** The main root meristematic zone (within approximately 80 μm from the root tip) of *Arabidopsis* seedlings was used as the test area. **(C)** Net Ca^2+^ fluxes at the meristematic zone in response to 300 mM mannitol applied as indicated by the arrow. Positive values indicate Ca^2+^ efflux. **(D)** Mean Ca^2+^ flux values from 1 to 5 min after 300 mM mannitol treatment and the control (pre 3 min) (*n* = 5). Different letters indicate significant differences with each treatment at *p*<0.05 level.

As AtANN1 mediates Ca^2+^ transport across the plasma membrane (Mohammad-Sidik et al., 2021), we examined the transmembrane Ca^2+^ flow in *AtANN2*- and *AtANN3-*related mutants and transgenic plants using an NMT system (Figure 5A). The main root meristematic zone (within approximately 80 μm from the root tip) of *Arabidopsis* seedlings was used as the test area (Figure 5B). To eliminate the error caused by the instability of Ca^2+^ flow within 1 min after mannitol treatment, the mean values were calculated for 3 min before (pre 3 min) and 1–5 min after the treatment (Figure 5D). No difference was detected in the mean values of Ca^2+^ flow for all tested genotypes before 3 min of treatment. Mannitol treatment caused Ca^2+^ efflux from the root tips. The function of AtANN2 was manifested under short-day condition: the activation of Ca^2+^ efflux from the root tip in *atann2* was weakened after mannitol treatment relative to that in Col-0, and *AtANN2-COM* did not differ from Col-0. The Ca^2+^ efflux was activated to a greater extent in *AtANN2-OE*. In contrast, the treatment effects on the related mutants of *AtANN2* were not observed under long-day condition (Figures 5C,D).

Unlike AtANN2, the function of AtANN3 manifested under long-day condition, but not short-day condition, as follows: the activation of Ca^2+^ efflux from the root tip in *atann3* was weakened after mannitol treatment relative to that in Col-0 and *AtANN3-COM*, while Ca^2+^ efflux was activated to a greater extent in *AtANN3-OE* (Figures 5C,D). Therefore, these results suggested that AtANN2 and AtANN3 significantly affected Ca^2+^ flow across the plasma membrane, and these functions manifested only under specific photoperiodic conditions.

### AtCRY2 Interacts With the AtANN2 and AtANN3 Proteins

**FIGURE 6:**
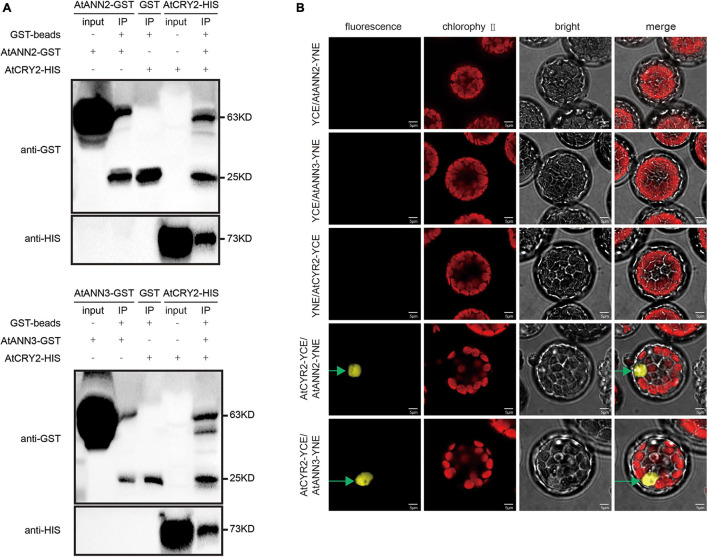
AtCRY2 interacts with AtANN2 and AtANN3 proteins. **(A)** GST pull-down assay. AtANN2-GST, AtANN3-GST, GST, and AtCRY2-HIS proteins were expressed in *Escherichia coli*. Purified proteins were used for the pull-down assay. Proteins immunoprecipitated were analyzed with anti-GST antibodies or anti-HIS antibodies. **(B)** Bimolecular fluorescence complementation (BiFC) assay: The C-terminal domain of YFP fused to AtCRY2 was coexpressed (AtCRY2-YCE) with a fusion protein of the N-terminal domain of YFP and AtANN2 or AtANN3 (AtANN2-YNE or AtANN3-YNE). YNE or YCE vectors expressing split YFP domains alone were used as controls. The constructs were co-injected into *Arabidopsis* leaf cells, and the YFP signals were observed after 48–72 h in transiently transformed protoplasts by fluorescence microscopy. Scale bar = 5 μm.

As the functions of AtANN2 and AtANN3 were regulated by photoperiod, we investigated whether the photoreceptor AtCRY2 could interact with the AtANN2 and AtANN3 proteins. By the pull-down approach to detect protein-protein interactions *in vitro*, AtANN2-GST was found to interact with AtCRY2-HIS, while AtANN3-GST interacted with AtCRY2-HIS (Figure 6A). The *in vivo* interaction was further revealed by bimolecular fluorescence complementation (BiFC) analysis (Figure 6B). AtCRY2-YCE could interact with AtANN2-YNE and AtANN3-YNE, which again demonstrated the interaction of AtCRY2 with AtANN2 and AtANN3 in *Arabidopsis* protoplasts. Based on these biochemical evidence, AtCRY2 could interact with AtANN2 and AtANN3 in plants and might play a role in the signaling pathway associated with the photoperiodic regulation of AtANN2 and AtANN3.

### Function of AtCRY2 in the Photoperiodic Regulation of AtANN2 and AtANN3 in Response to Drought Stress

**FIGURE 7:**
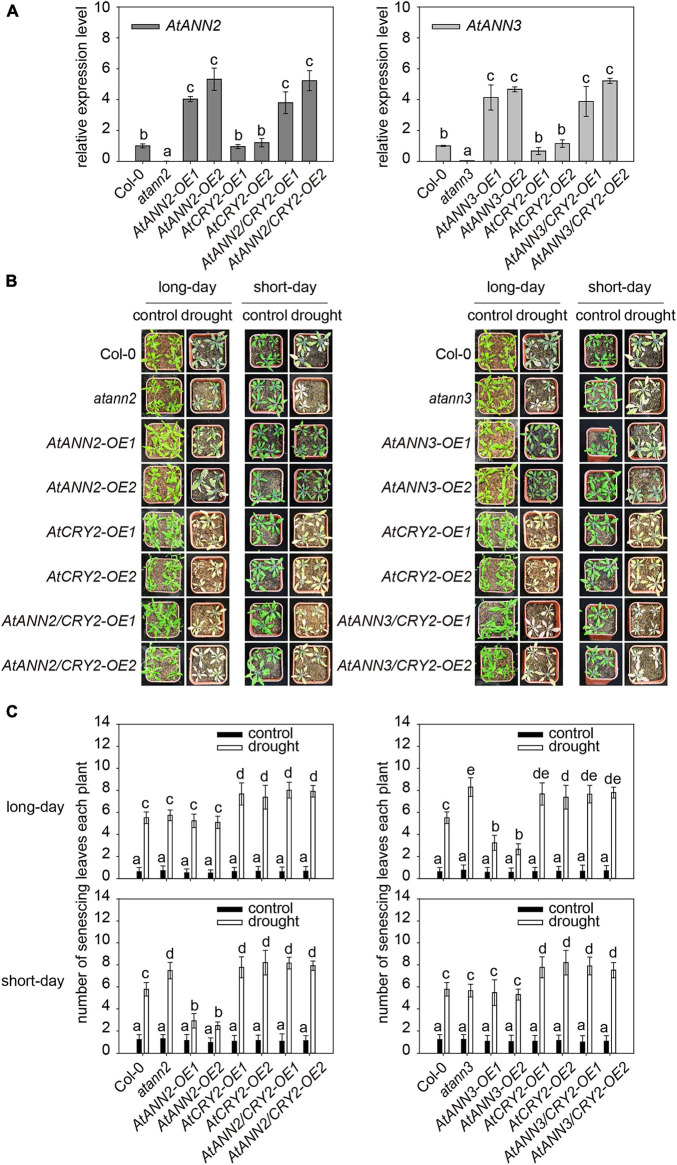
Function of AtCRY2 in the photoperiodic regulation of AtANN2 and AtANN3 in response to drought stress. **(A)** Relative expression of *AtANN2* and *AtANN3* genes in the *AtCRY2-OE* background (*AtANN2*/*CRY2-OE* and *AtANN3/CRY2-OE*) and those in the Col-0 background (*AtANN2*-*OE* and *AtANN3-OE*). **(B)** Plant growth phenotype (senescing leaves) of the *AtCRY2*-related lines the same as Figure 7A in response to drought stress under long- or short-day conditions. **(C)** Statistical analysis of senescing leaves of genotypes in Figure 7B. Different letters indicate significant differences with each treatment at *P*<0.05 level.

AtCRY2 has been found to play a negative regulatory role in drought stress, with the *cry1cry2* double mutants showing better growth under drought stress. The *cry2* single mutant showed a similar phenotype as WT, and *AtCRY2-OE* has more sensitive to blue light regulation of stomatal opening (Mao et al., 2005). To test whether the function of AtCRY2 in drought results from its regulation on AtANN2 and AtANN3, we generated transgenic lines overexpressing the *AtANN2* and *AtANN3* genes in the *AtCRY2-OE* background (*AtANN2*/*CRY2-OE* and *AtANN3/CRY2-OE*). The enhanced expression levels of *AtANN2* and *AtANN3* in the *AtCRY2-OE* background (3∼6-fold) did not differ from those in the Col-0 background (Figure 7A).

Overexpression of *AtANN2* in Col-0 enhanced drought tolerance as revealed by less number of senescing leaves (2.48–2.92) under short-day condition (Figures 1C,D). However, the physiological contribution of the overexpressed AtANN2 protein was not observed in the *AtCRY2-OE* background with the number of senescing leaves (7.92–8.16) (Figures 7B,C). This finding indicated that overexpression of *AtCRY2* could negatively regulate the function of AtANN2 in drought tolerance under short-day condition. The same negative regulation of AtANN3 under long-day condition was also shown in transgenic lines overexpressed *AtANN3* in the *AtCRY2-OE* background with the number of senescing leaves (7.66–7.80) compared to those in the Col-0 background (2.66–3.24) (Figures 7B,C). Thus, these results indicated that the function of AtANN2 and AtANN3 in drought tolerance could be repressed by *AtCRY2* overexpression under specific photoperiods.

### AtCRY2 Regulated the Subcellular Location of AtANN2 and AtANN3 in Response to Mannitol Treatment

**FIGURE 8:**
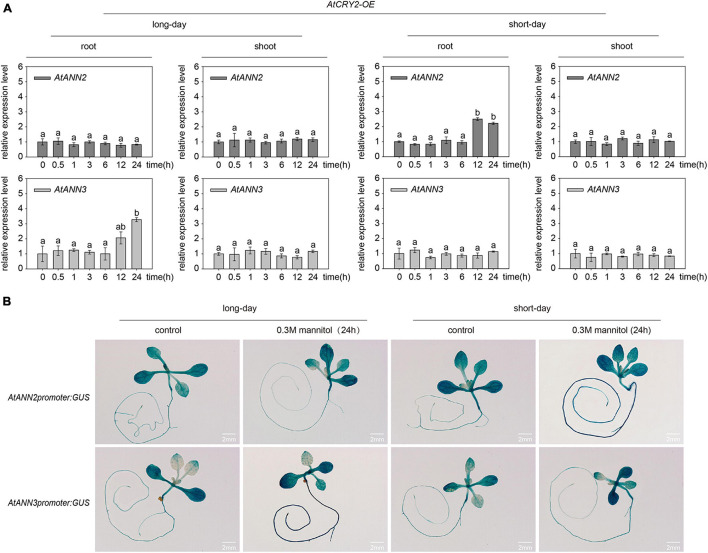
AtCRY2 did not regulated gene expression of *AtANN2* and *AtANN3* in response to mannitol treatment. **(A)** Relative expression levels of *AtANN2* and *AtANN3* in 300 mM mannitol-treated plants (*AtCRY2-OE*) for 0, 0.5, 1, 3, 6, 12, and 24 h using qRT-PCR. Different letters indicate significant differences with each treatment at *P*<0.05 level. **(B)** Induction of *GUS* expression in *AtCRY2-OE* roots in response to 300 mM mannitol treatment for 24 h under different photoperiods. Scale bar = 2 mm.

To understand how the overexpression of *AtCRY2* affected the functions of AtANN2 and AtANN3, we first investigated the gene expression patterns of *AtANN2* and *AtANN3* in the *AtCRY2-OE* lines by qRT-PCR. After mannitol treatment for 12 and 24 h, the expression of *AtANN2* in roots was increased up to 2.22- to 2.50-fold under short-day condition, and the expression of *AtANN3* was increased up to 2.06- to 3.28-fold under long-day condition (Figure 8A). These patterns were further confirmed by transgenic lines expressing the corresponding *GUS* reporter genes for *AtANN2* and *AtANN3* (Figure 8B). The expression levels of *AtANN2* and *AtANN3* in *AtCRY2-OE* were similar to that in Col-0 (Figure 3), indicating that *AtCRY2* did not regulate *AtANN2* and *AtANN3* expression at the transcriptional level.

**FIGURE 9:**
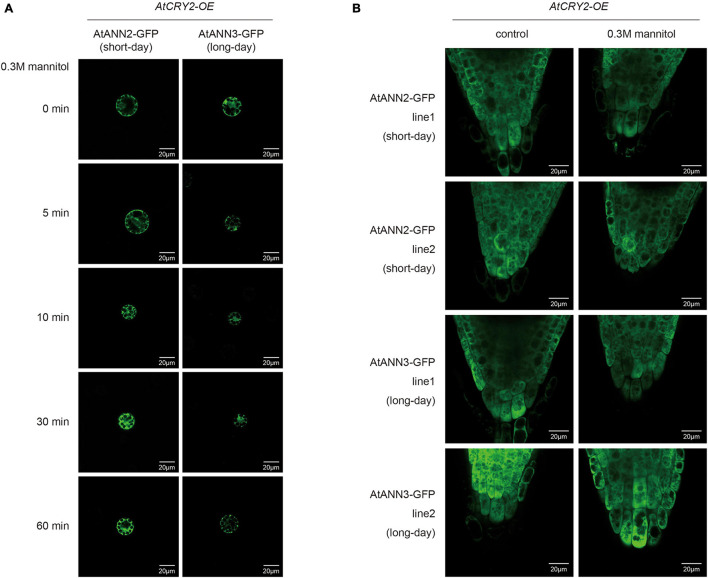
AtCRY2 regulated subcellular location of AtANN2 and AtANN3 in response to mannitol treatment. **(A)** Subcellular localization of the AtANN2-GFP and AtANN3-GFP fusion proteins in response to mannitol treatment for 0, 5, 10, 30, and 60 min using leaf cell protoplasts of *AtCRY2-OE* plants grown under different photoperiodic conditions. Scale bar = 20 μm. **(B)** Subcellular localization of AtANN2-GFP and AtANN3-GFP fusion proteins using the transgenic lines in the *AtCRY2-OE* background subjected to treatment the same as Figure 4B.

We then investigated changes in the subcellular localization of AtANN2 and AtANN3 by *AtCRY2* overexpression. After mannitol treatment, unlike that in Col-0 (Figure 4A and Supplementary Figure 2A), neither AtANN2-GFP nor AtANN3-GFP was able to target to the plasma membrane within 60 min in the protoplast of *AtCRY2-OE* (Figure 9A and Supplementary Figure 3A) irrespective of long-day or short-day conditions. By analysis of transgenic lines expressing *AtANN2-GFP* and *AtANN3-GF*P in *AtCRY2-OE* background under control of their native promoters, it revealed that the subcellular localization of AtANN2-GFP and AtANN3-GFP appeared no longer at the plasma membrane after 5 min of mannitol treatment (Figure 9B and Supplementary Figure 3B) as they did in Col-0 background (Figure 4B and Supplementary Figure 2B). Thus, AtCRY2 was proposed to repress function of AtANN2 and AtANN3 by affecting their plasma membrane localization in response to mannitol treatment.

### AtCRY2 Inhibited AtANN2- and AtANN3-Dependent Transmembrane Ca^2+^ Flow

**FIGURE 10:**
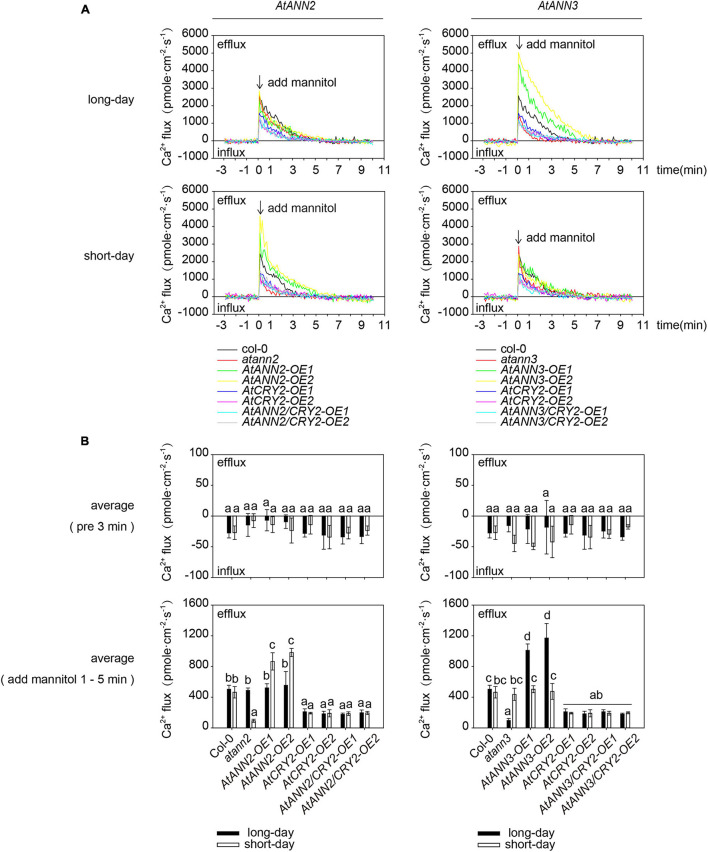
AtCRY2 inhibited AtANN2- and AtANN3-dependent transmembrane Ca^2+^ flow. **(A)** Transmembrane Ca^2+^ flow analysis in transgenic lines overexpressing *AtANN2* and *AtANN3* either in the Col-0 (*AtANN2-OE* and *AtANN3-OE*) or the *CRY2-OE* background (*AtANN2/CRY2-OE* and *AtANN3/CRY2-OE*) subjected to treatment the same as Figure 5C. **(B)** Mean Ca^2+^ flux values from 1 to 5 min after 300 mM mannitol treatment and control (pre 3 min) (*n* = 5) in Figure 10A. Different letters indicate significant differences with each treatment at *p*<0.05 level.

To test whether AtCRY2 affected the calcium channel activity of AtANN2 and AtANN3 in the drought signaling pathway, the transmembrane Ca^2+^ flow analysis was performed in transgenic lines overexpressing *AtANN2* and *AtANN3* either in Col-0 (*AtANN2-OE and AtANN3-OE*) or *CRY2-OE* background (*AtANN2/CRY2-OE* and *AtANN3/CRY2-OE*). Unlike that of *AtANN2-OE* and *AtANN3-OE*, mannitol activation of Ca^2+^ efflux was diminished in *AtANN2/CRY2-OE* under short-day condition and in *AtANN3/CRY2-OE* under long-day condition (Figure 10). This result suggested that AtCRY2 could negatively regulate the ion flow across the plasma membrane mediated by AtANN2 and AtANN3.

## Discussion

**FIGURE 11:**
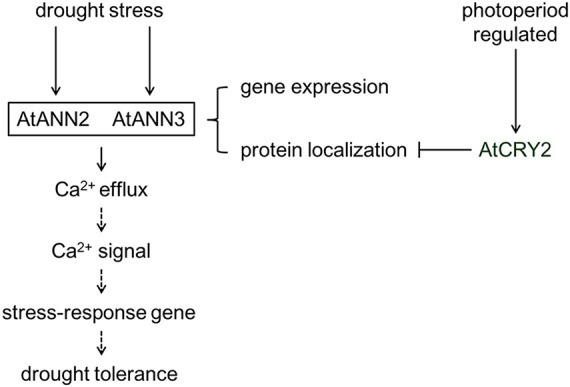
Working model of the regulation of AtANN2 and AtANN3 in drought tolerance by photoperiod. The functions and subcellular localizations of AtANN2 and AtANN3 in drought tolerance were dependent on the photoperiod. Under normal conditions, AtANN2- and AtANN3-dependent GFP signals were widely expressed within the cells, not only at the plasma membrane. In response to drought stress, they could target to the plasma membrane for a short time (5 min) and acted as Ca^2+^ channels to caused Ca^2+^ efflux. Their gene expression was also changed after drought treatment by longer time (24 h) signals. Cytoplasmic free calcium ([Ca^2+^]_cyt_) might alter triggered calcium signaling. Calcium signaling caused downstream expression of genes associated with drought stress and thus adaptation to environmental changes. AtCRY2 is proposed to repress functions of AtANN2 and AtANN3 by affecting their plasma membrane localization to inhibited AtANN2 and AtANN3-dependent transmembrane Ca^2+^ flow in response to drought stress.

The plasma membrane serves as the interface between the cell and the environment for exchange of nutrients and signals. Annexins can act as sensors sensing calcium signals early in the environmental signaling pathway and can also trigger the generation of specific downstream calcium signals. The study of the physiological functions, environmental regulators, and signal transduction of annexins in plant cells is currently in its infancy. In this study, we found that photoperiod regulates the physiological function, protein localization, and calcium channel activity of AtANN2 and AtANN3. AtCRY2 is proposed to repress the functions of AtANN2 and AtANN3 by affecting their plasma membrane localization and inhibiting AtANN2- and AtANN3-dependent transmembrane Ca^2+^ flow in response to drought stress (Figure 11). This study has theoretical significance for elucidating the physiological functions and activity regulation mechanisms of annexins.

### AtANN2 and AtANN3 Were Involved in Drought Stress Processes and Regulated by Photoperiod

The functions of AtANN1 and AtANN4 depended on the photoperiod implied that the physiological functions of annexins might change under different environmental signals, such as different photoperiods. Konopka-Postupolska et al. (2009) found that *atann1* was more sensitive to drought stress under short-day condition relative to Col-0. However, Huh et al. (2010) found that under long-day condition, AtANN1 and AtANN4 interacted, and *atann1*/*ann4* was more resistant to drought stress than *atann1* or *atann4*. The mechanism by which light signals regulated annexins’ function and thus affect plant stress tolerance response is currently unknown. In this study, the physiological functions of AtANN2 and AtANN3 were examined under the same experimental system, which were found to have a positive regulatory effect on drought stress tolerance, with AtANN2-related genotypes showed different responses to drought stress under short-day condition, while a role of AtANN3 revealed in drought tolerance under long-day condition. These results indicated that the functions of AtANN2 and AtANN3 in drought tolerance depended on the photoperiod (Figure 1).

### The Physiological Functions of AtANN2 and AtANN3 Under Photoperiodic Regulation Were Closely Related to Their Expression and Localization

Eight members of the *Arabidopsis* annexin family were highly expressed at the seed germination and seedling stages. In this study, we also found that *AtANN2 and AtANN3* expressed mainly in roots and cotyledons of 3-day-old seedlings, slightly increased in root tips and shoots of 7-day-old seedlings (Figure 2B). Cantero et al. (2006) also found that *AtANN*2 was mainly expressed in root epidermal cells and lateral root primordium cells, and *AtANN2* was assumed to play a role in root responses to external signals. In this study, we found that the photoperiod regulated the expression patterns of *AtANN2* and *AtANN3* in roots responded to mannitol treatment (Figure 3). Other studies have also found that light signals can regulate the expression of other annexin genes as follows (Baucher et al., 2011): *AtANN1* and *AtANN4* were regulated by *AtHY5* (Lee et al., 2007). *AtANN5* expression was increased in hypocotyls by red light irradiation, and far-red light reversed this response (Cantero et al., 2006). *Annexin* gene expression in tobacco (*Nicotiana tabacum*) was also regulated by light signals as follows: gene expression was evident in roots under light conditions, but no expression in roots was observed under dark conditions (Baucher et al., 2012).

Protein level regulation enabled a more rapid response to external signals and changed in function through regulation of protein activity and localization. The subcellular localization of proteins was closely related to the functions of annexins, and the same annexin could be localized in multiple regions in the cell (Laohavisit and Davies, 2009). This study found that under normal conditions AtANN2- and AtANN3-dependent GFP signals were widely expressed within the cells, not only at the plasma membrane. However, in response to mannitol treatment, the AtANN2 and AtANN3 proteins could be targeted to the plasma membrane, and this regulatory process was regulated by photoperiod (Figure 4). Other researchers have also found similar phenomena, such as AtANN1 translocation from the cytoplasm to the plasma membrane in root cells under salt stress (Lee et al., 2004). Wheat (*Triticum aestivum*) annexins under cold stress started to aggregate at the plasma membrane and functioned as calcium channels (Breton et al., 2000). Annexins are localized in other subcellular structures, e.g., the nucleus, vesicle membrane, and Golgi apparatus (Konopka-Postupolska and Clark, 2017). Different locations may be associated to different functions and regulatory proteins, and whether AtANN2- and AtANN3-regulated functions are related to other subcellular organelles, such as the nuclei, requires further investigation.

Plants receive drought signals, initiate regulation at the transcriptional level after a long period, and induce changes in subcellular localization within a short period. A multilayered pattern of regulation helps plants adapt to changing environments faster with less consumption.

### AtANN2 and AtANN3 Might Cause Downstream Calcium Signaling by Affecting Ca^2+^ Flow Across the Plasma Membrane Under Specific Photoperiodic Conditions

In response to mannitol treatment, the AtANN2 and AtANN3 proteins could be targeted to the plasma membrane, and the altered subcellular localization was closely related to their protein functions. Certain annexins localized in the plasma membrane are currently known to be closely related to transmembrane ion transport, such as AtANN1, which constructs the root epidermal cell plasma membrane Ca^2+^ channel (Laohavisit et al., 2012; Richards et al., 2014; Zhao et al., 2019; Mohammad-Sidik et al., 2021). On the other hand, the ability of annexins to regulate ion transport may be related to their regulated resistance responses, for example, AtANN1 acted as a root plasma membrane calcium channel during salt, hyperosmotic, oxidative, and cold stress exposure by regulating the Ca^2+^ concentration in response to external changes (Laohavisit et al., 2012, 2013; Richards et al., 2014; Liu et al., 2021). AtANN4, which is transiently expressed in *Xenopus* (*Xenopus laevis*) oocytes, could also be embedded in the plasma membrane to caused calcium signaling in response to salt stress (Ma et al., 2019). Most previous studies have used membrane clamp techniques to investigate ion flow in transiently expressed *Xenopus* oocytes and plant protoplasts. In this study, the transmembrane Ca^2+^ flow in plant roots was examined using the NMT system (Figure 5A). This *in vivo* assay technique allows the detection of the rate and direction of ion flows in and out of living organisms without damaging roots. We found that AtANN2 and AtANN3 significantly affected Ca^2+^ flow across the plasma membrane, and these functions manifested only under specific photoperiodic conditions. Specifically, the function of AtANN2 was manifested under short-day condition, while the function of AtANN3 manifested under long-day condition (Figure 5). In addition to annexins, calcium signaling induced by stress signals involved multiple types of ion channels and signal transduction systems that together regulated local calcium signaling or long-distance calcium signaling. For example, AtCNGC19 could be rapidly activated as a Ca^2+^ channel in wounding stress, caused Ca^2+^ influx (Meena et al., 2019).

### AtCRY2 Plays a Negative Regulatory Role in the AtANN2 and AtANN3 Signaling Pathways by Photoperiod

We found that the function of AtANN2 was manifested under short-day condition, while the function of AtANN3 manifested under long-day condition. How did annexins sense photoperiods? Photoreceptors sensed photoperiod and thus regulated plant growth, development, and stress responses (Yoshitake et al., 2015; Fantini and Facella, 2020), e.g., PHYs have been found to be involved in photoperiod sensing in short-day plant flowering. In long-day plants, both PHYs and CRYs were involved in photoperiod perception (Su et al., 2017; Fantini et al., 2019). Therefore, annexins were speculated to also sense photoperiods through photoreceptors. We found that AtCRY2 interacted with AtANN2 and AtANN3 (Figure 6).

On the other hand, CRYs were also involved in biotic and abiotic stresses, such as drought stress, salt stress, heat stress, low temperature stress, radiation, UV-B light, and pathogen attacks (Mao et al., 2005; Ahmad, 2016; Zhou et al., 2017, 2018). Most recent studies only focused on phenotypes, such as CRYs increased the sensitivity of plants to drought stress (D’Amico-Damiao and Carvalho, 2018; Aslam et al., 2020). Overexpression of CRYs in *wheat* and *barley* (*Hordeum vulgare*) resulted in worse growth status in *Arabidopsis* (Mao et al., 2005; Xu et al., 2009; Zhou et al., 2018). Knowledge regarding the mechanism is limited. We found that after *AtCRY2* overexpression, the drought tolerance functions of AtANN2 and AtANN3 under a specific photoperiod were inhibited (Figure 7), their subcellular localization no longer appeared at the plasma membrane after 5 min of mannitol treatment (Figure 9), and mannitol activation of Ca^2+^ efflux was diminished (Figure 10). These results suggest that AtCRY2 is proposed to repress functions of AtANN2 and AtANN3 by affecting their plasma membrane localization to inhibited AtANN2 and AtANN3-dependent transmembrane Ca^2+^ flow in response to drought stress.

Taken together, these results demonstrated the existence of a “photoperiod - AtCRY2 -annexins (AtANN2 and AtANN3) - transmembrane Ca^2+^ flow - drought tolerance function” signaling pathway in *Arabidopsis*. This finding highlights a novel mechanism regulating the drought stress response by light signal transduction. Further research is needed to determine the role of the biological clock in regulating this signaling pathway and their downstream genes involved in drought tolerance.

## Data Availability Statement

The original contributions presented in the study are included in the article/Supplementary Material, further inquiries can be directed to the corresponding author.

## Author Contributions

TL conceived the experiments and drafted the manuscript. TL, LD, QL, JK, QG, and SW participated in the main experiments. LD and JK analyzed the data in this work. QL helped to revise the manuscript. All authors read and approved the final manuscript.

## Conflict of Interest

The authors declare that the research was conducted in the absence of any commercial or financial relationships that could be construed as a potential conflict of interest.

## Publisher’s Note

All claims expressed in this article are solely those of the authors and do not necessarily represent those of their affiliated organizations, or those of the publisher, the editors and the reviewers. Any product that may be evaluated in this article, or claim that may be made by its manufacturer, is not guaranteed or endorsed by the publisher.

## Supplementary Material

Supplementary Figure 1 The ratio of plants in response to mannitol treatment by regulating subcellular localization. Subcellular localization of AtANN2-GFP and AtANN3-GFP fusion proteins using the transgenic lines. 10-day-old plants under different photoperiods were treated with 300 mM mannitol for 5 min, and GFP signals were observed under a confocal laser-scanning microscope. At least 10 plants were counted in each treatment. Plasma membrane signal (%) = number of plants responding to mannitol treatment/number of total plants.

Supplementary Figure 2 Regulation of the subcellular localization of AtANN2 and AtANN3 in response to mannitol treatment under different photoperiods. **(A)** Subcellular localization of the AtANN2-GFP and AtANN3-GFP fusion proteins in response to mannitol treatment for 0, 5, 10, 30, and 60 min using leaf cell protoplasts of Col-0 plants grown under different photoperiodic conditions. Scale bar = 20 μm. **(B)** Subcellular localization of AtANN2-GFP and AtANN3-GFP fusion proteins using the transgenic lines. 10-day-old plants under different photoperiod were treated with 300 mM mannitol for 5 min and GFP signals were observed under a confocal laser-scanning microscope. Scale bar = 20 μm.

Supplementary Figure 3 AtCRY2 regulated subcellular location of AtANN2 and AtANN3 in response to mannitol treatment. **(A)** Subcellular localization of the AtANN2-GFP and AtANN3-GFP fusion proteins in response to mannitol treatment for 0, 5, 10, 30, and 60 min using leaf cell protoplasts of *AtCRY2-OE* plants grown under different photoperiodic conditions. Scale bar = 20 μm. **(B)** Subcellular localization of AtANN2-GFP and AtANN3-GFP fusion proteins using the transgenic lines in the *AtCRY2-OE* background subjected to treatment the same as Supplementary Figure 2B.
