# Supplementary figures and images for: AtCRY2 Negatively Regulates the Functions of AtANN2 and AtANN3 in Drought Tolerance by Affecting Their Subcellular Localization and Transmembrane Ca2+ Flow
Source: Front Plant Sci. 2021 Nov 23;12:754567. doi: 10.3389/fpls.2021.754567 (PMC8649957; doi:10.3389/fpls.2021.754567)

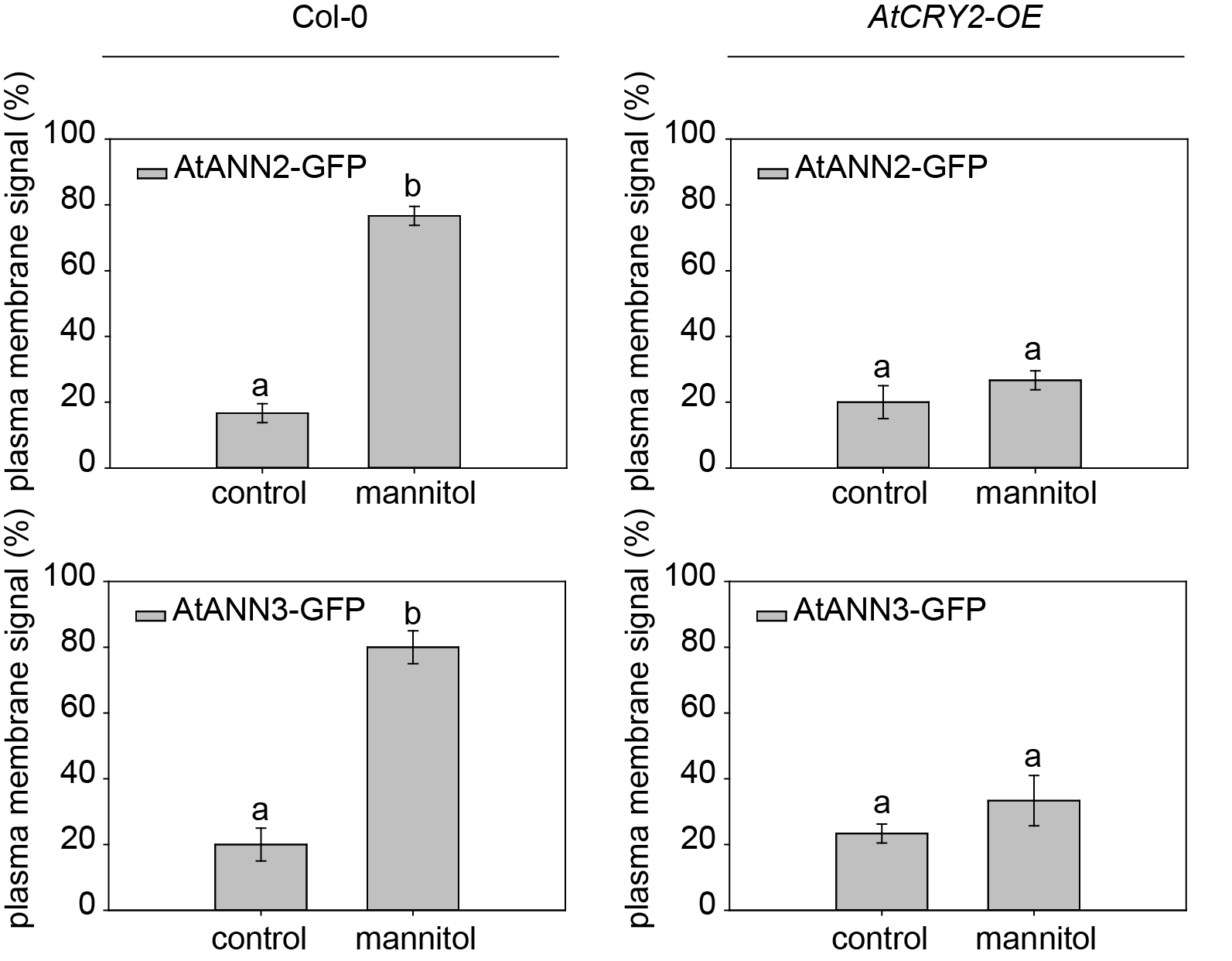

Supplement: Supplementary Figure 1 — The ratio of plants in response to mannitol treatment by regulating subcellular localization. Subcellular localization of AtANN2-GFP and AtANN3-GFP fusion proteins using the transgenic lines. 10-day-old plants under different photoperiods were treated with 300 mM mannitol for 5 min, and GFP signals were observed under a confocal laser-scanning microscope. At least 10 plants were counted in each treatment. Plasma membrane signal (%) = number of plants responding to mannitol treatment/number of total plants. [file Image_1.TIF]

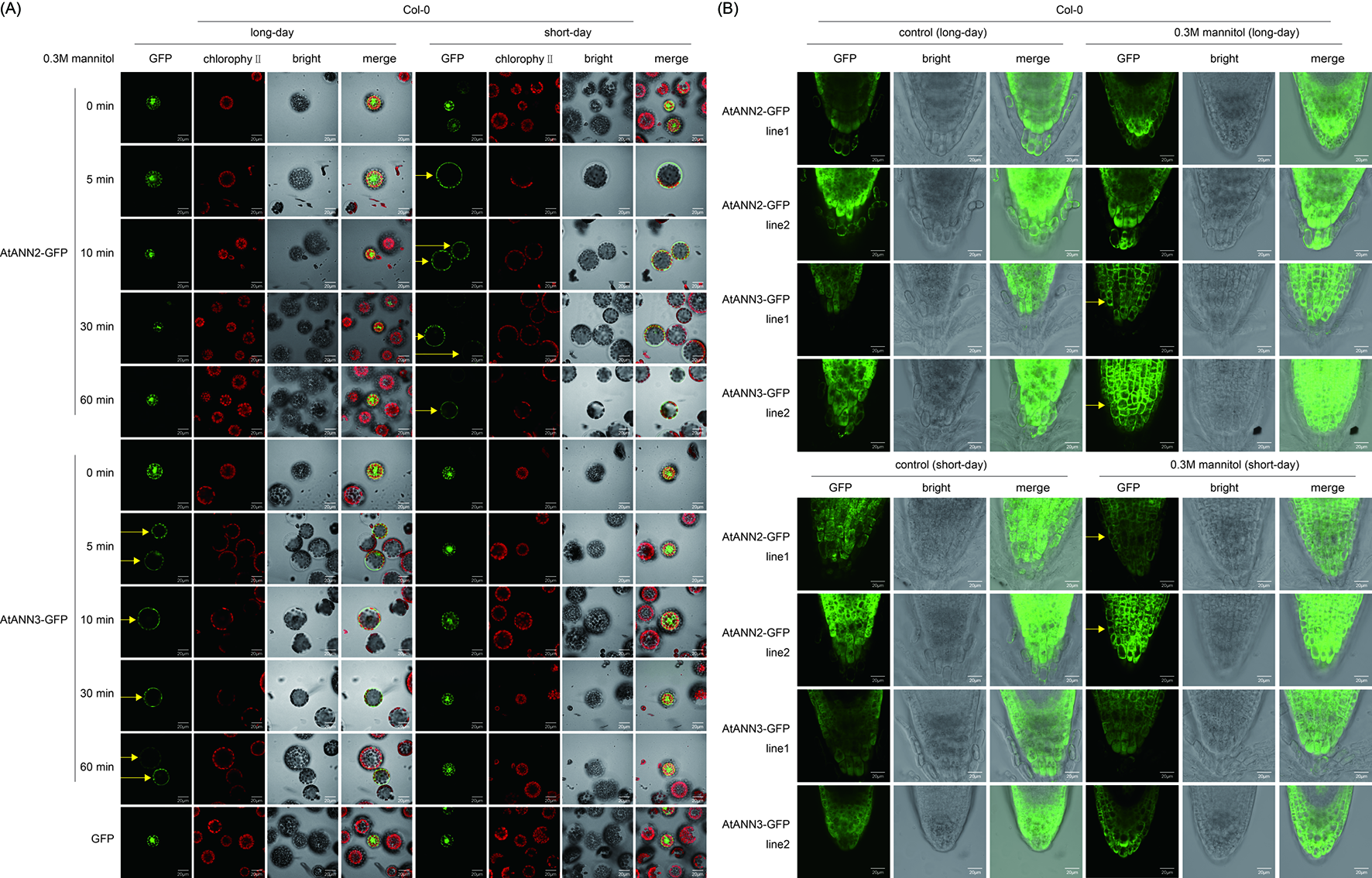

Supplement: Supplementary Figure 2 — Regulation of the subcellular localization of AtANN2 and AtANN3 in response to mannitol treatment under different photoperiods. (A) Subcellular localization of the AtANN2-GFP and AtANN3-GFP fusion proteins in response to mannitol treatment for 0, 5, 10, 30, and 60 min using leaf cell protoplasts of Col-0 plants grown under different photoperiodic conditions. Scale bar = 20 μm. (B) Subcellular localization of AtANN2-GFP and AtANN3-GFP fusion proteins using the transgenic lines. 10-day-old plants under different photoperiod were treated with 300 mM mannitol for 5 min and GFP signals were observed under a confocal laser-scanning microscope. Scale bar = 20 μm. [file Image_2.TIF]

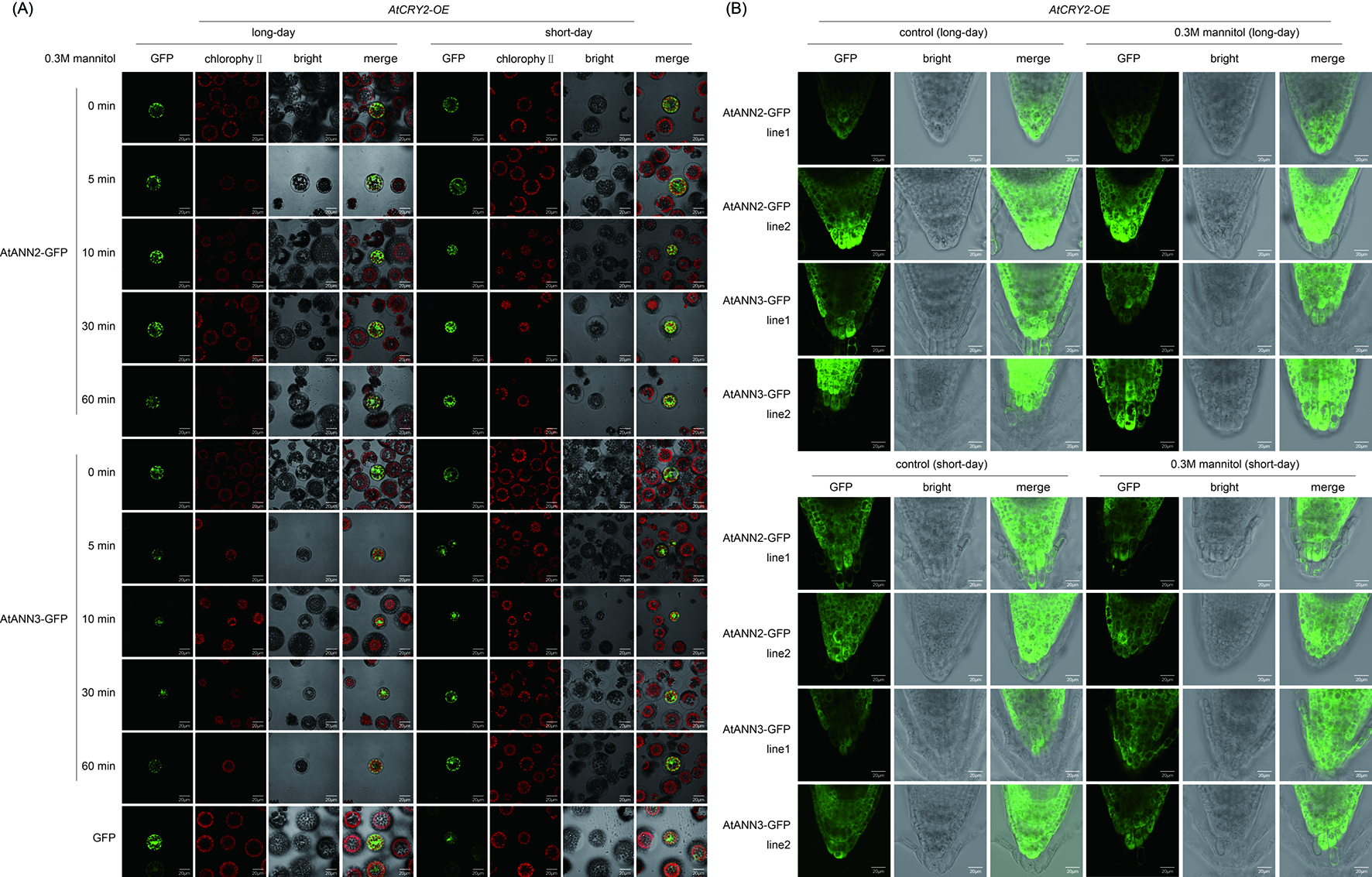

Supplement: Supplementary Figure 3 — AtCRY2 regulated subcellular location of AtANN2 and AtANN3 in response to mannitol treatment. (A) Subcellular localization of the AtANN2-GFP and AtANN3-GFP fusion proteins in response to mannitol treatment for 0, 5, 10, 30, and 60 min using leaf cell protoplasts of AtCRY2-OE plants grown under different photoperiodic conditions. Scale bar = 20 μm. (B) Subcellular localization of AtANN2-GFP and AtANN3-GFP fusion proteins using the transgenic lines in the AtCRY2-OE background subjected to treatment the same as Supplementary Figure 2B. [file Image_3.TIF]
